# RURAL WOMEN AND AGING: IMPLICATIONS FOR WORK AND RETIREMENT OF OLDER WOMEN

**DOI:** 10.1093/geroni/igz038.065

**Published:** 2019-11-08

**Authors:** Aine Ni Leime, Nata Duvvury, Tanya Watson

**Affiliations:** National University of Ireland, Galway, Galway, Galway, Ireland

## Abstract

At least half of the world’s female population live in rural areas, and many are ageing. For these rural women, agriculture and informal rural livelihoods are the primary sources of employment, posing critical challenges for them with regard to work and retirement. This paper focuses on the interaction between the twin phenomena of the feminisation of agriculture and the feminisation of ageing and the consequent implications for rural women’s work and retirement. Drawing on qualitative interviews and focus groups with 48 older rural Irish women, the paper establishes the ‘invisibility’ of women’s economic contribution in agriculture, limiting their pension accumulation and constraining their retirement planning. The study found that even women property owners, and designated ‘farmers’, had uncertainty about their pension or retirement income. A key conclusion is that rural women’s pension rights are still not guaranteed posing increased risk of economic insecurity and wellbeing for older rural women.

